# Quantitative Fundus Autofluorescence in HCQ Retinopathy

**DOI:** 10.1167/iovs.61.11.41

**Published:** 2020-09-25

**Authors:** Vivienne C. Greenstein, Jose Ronaldo Lima de Carvalho, Rait Parmann, Luz Amaro-Quireza, Winston Lee, Donald C. Hood, Stephen H. Tsang, Janet R. Sparrow

**Affiliations:** 1 Department of Ophthalmology, Columbia University Medical Center, New York, New York, United States; 2 Department of Ophthalmology, New York University School of Medicine, New York, New York, United States; 3 Deparment of Psychology, Columbia University, New York, New York, United States; 4 Department of Pathology & Cell Biology, Columbia University Medical Center, New York, New York, United States

**Keywords:** hydroxychloroquine, near-infrared fundus autofluorescence, photoreceptor cells, Plaquenil, quantitative fundus autofluorescence, retina, retinal pigment epithelium, short-wavelength fundus autofluorescence

## Abstract

**Purpose:**

To increase our understanding of the mechanisms underlying hydroxychloroquine (HCQ) retinopathy, analyses by quantitative fundus autofluorescence (qAF) and near-infrared fundus autofluorescence (NIR-AF) were compared to results obtained by recommended screening tests.

**Methods:**

Thirty-one patients (28 females, 3 males) were evaluated with standard automated perimetry and spectral domain optical coherence tomography (SD-OCT); 28 also had multifocal electroretinography (mfERG). Measurement of short-wavelength fundus autofluorescence (SW-AF) by qAF involved the use of an internal fluorescent reference and intensity measurements in eight concentric segments at 7° to 9° eccentricity. For semiquantitative analysis of NIR-AF, intensities were acquired along a vertical axis through the fovea.

**Results:**

Four of 15 high-dose (total dose >1000 g, daily dose >5.0 mg/kg) patients and one of 16 low-dose (total dose <1000 g, daily dose 4.4 mg/kg) patients were diagnosed with HCQ-associated retinopathy based on abnormal 10-2 visual fields, SD-OCT, and SW-AF imaging. Three of the high-dose patients also had abnormal mfERG results. Of the five patients exhibiting retinopathy, two had qAF color-coded images revealing higher intensities inferior, nasal, and lateral to the fovea. The abnormal visual fields also exhibited superior-inferior differences. Mean NIR-AF gray-level intensities were increased in four high-dose patients with no evidence of retinopathy. In two patients with retinopathy, NIR-AF intensity within the parafovea was below the normal range. One high-dose patient (6.25 mg/kg) had only abnormal mfERG results.

**Conclusions:**

These findings indicate that screening for HCQ retinopathy should take into consideration superior-inferior differences in susceptibility to HCQ retinopathy.

Hydroxychloroquine (HCQ), an antimalarial drug, is widely used in the treatment of various autoimmune diseases, including rheumatoid arthritis, systemic lupus erythematosus, and systemic sclerosis.^1^ Chloroquine (CQ), which is 10-fold more toxic to the retinal pigment epithelium (RPE), is also now employed to treat some cases of discoid lupus.^2^

An ocular side effect of long-term HCQ therapy is toxic retinopathy. With progression of the retinopathy, a “bull's-eye” maculopathy (BEM) develops. The BEM is visible in short-wavelength fundus autofluorescence (SW-AF) (488 nm) and near-infrared fundus autofluorescence (NIR-AF) (787 nm) images as a paracentral ring of hyperautofluorescence with sparing of the fovea.^3^^,^^4^ With more advanced disease, the pericentral retina becomes mottled with increased SW-AF in the adjacent peripheral retinal area. In Asian patients, the pericentral pattern is more typical.^5^^,^^6^ HCQ retinopathy is infrequent, but the risk is dependent on daily dose and duration of treatment. The risk is increased if the daily dose is greater than 5.0 mg/kg and duration is longer than 5 years.^7^^,^^8^ The prevalence of toxicity is reported to be approximately 7.5% after 5 years of treatment.^7^ It is irreversible and can progress even after cessation of therapy.^9^^–^^11^

Patients being treated with HCQ require monitoring for retinopathy. Specific recommendations for screening for retinopathy have recently been revised.^7^ In addition to an ophthalmologic examination, the recommended primary screening tests are automated “white-on-white” 10-2 visual fields (with 24-2/30-2 visual fields for Asian patients) and spectral domain-optical coherence tomography (SD-OCT). The recommendations also include one or more of the following objective tests: the multifocal electroretinogram (mfERG), as it provides objective corroboration for visual fields, and SW-AF, as it provides a topographic view of damage across the posterior pole.^7^ It has been reported that abnormalities in the SD-OCT scans may be detected before visual field defects.^12^ While other investigators consider SD-OCT to be less sensitive than visual field testing or mfERG, SD-OCT is definitive when regional thinning is present in a typical pattern. Moreover, loss of the external limiting membrane (ELM) in SD-OCT images is considered a sign of impending disease progression.^11^ Although functional abnormalities detected with mfERG and visual field testing have been reported to be more frequently observed than morphologic abnormalities in SW-AF images,^3^^,^^13^ the latter modality has the advantage of being more efficient, and it provides topographical information regarding the areas of retinal damage. The presence of retinal pigment epithelial damage in SW-AF images is considered a predictor of maculopathy.^14^ Patients with HCQ retinopathy also exhibit significantly prolonged mean fluorescence lifetimes within the parafoveal area.^15^

Early studies of CQ, the related drug, revealed that as a weak base, it accumulates in lysosomes.^16^ Based on research in animals and cellular assays, it was also suggested that HCQ toxicity begins in RPE cells.^17^^,^^18^ Since HCQ has an affinity for RPE lysosomes,^19^ the drug may interact with bisretinoid lipofuscin, which also accumulates in lysosomal storage bodies. These lipofuscin fluorophores form in photoreceptor cells but are transferred secondarily to RPE cells within phagocytosed outer segments. As such, bisretinoids constitute a cellular burden that distinguishes RPE. Since the bisretinoids that constitute the lipofuscin of retina are the source of SW-AF, one of our objectives in this study was to determine whether SW-AF intensities measured by quantitative fundus autofluorescence in HCQ-treated individuals exceed the range of healthy eyes.^20^ We also assessed patterns of quantitative fundus autofluorescence (qAF) and NIR-AF topography, relative to healthy eyes. To gain further insight into the underlying mechanisms of HCQ toxicity, we compared the results to those obtained using recommended screening tests.

## Methods

This was a retrospective observational study of 31 patients referred to the Department of Ophthalmology at Columbia University Medical Center by rheumatologists and ophthalmologists for evaluation of HCQ retinopathy. Inclusion criteria for the study were current or previous history of HCQ treatment together with clinic visits that included automated “white-on-white” visual fields, SD-OCT, and SW-AF imaging. For those patients who did not have BEM on ophthalmoscopy, mfERGs were also required. In 13 patients, NIR-AF images were also obtained. The 31 patients who fit the inclusion criteria included 28 women and 3 men who ranged in age from 10 to 78 years (mean 47 ± 16 years), had corrected visual acuity of 20/50 or better, and had no signs of ocular disease aside from HCQ retinal toxicity (see Table for details).

**Table.** Patient Demographics and Usage of HCQ

							BCVA		
ID	Gender	Age, y	HCQ Indication	Daily Dose, mg/kg	Duration, y	Total Dose, g	OD	OS	Race/Ethnicity
1	F	16	Lyme disease	3.7	0.5	37	20/20; 20/25		Caucasian
2	M	51	Psoriatric arthritis	5.7	9	1314	20/20; 2020		Caucasian
3	F	36	SLE	6.7	10	1460	20/20; 20/20		Caucasian
**4** ^*^	**F**	**58**	**SLE**	**5.6**	**20**	**2798**	20/25; 20/25		Caucasian
5	F	33	Sjögren syndrome	6.5	1.5	150	20/20; 20/20		Asian
6	F	53	SLE	1.5	7	256	20/20**^#^**		Asian
7	F	56	RA	2.9	0.2	15	20/30**^#^**		Caucasian
8	F	33	SLE	3.7	14	1176	20/20**^#^**		Hispanic
9	F	48	RA	5.6	6	468	20/20; 20/20		Caucasian
**10** ^*^	**F**	**50**	**SLE**	**8.9**	**10**	**1460**	20/25**^#^**		Caucasian
11	F	55	SLE	6.5	17	1971	20/20; 20/40		Caucasian
12	F	43	Mixed connective tissue disease	4.4	5	548	20/20; 20/20		Caucasian
13	M	58	Behcet syndrome	4.2	7	511	20/30; 20/25		Asian
**14** ^*^	**F**	**48**	**SLE**	**7.2**	**10**	**1128**	20/25**^#^**		Asian
**15** ^*^	**F**	**50**	**SLE**	**4.4**	**5**	**730**	20/25; 20/25		Caucasian
**16** ^*^	**F**	**49**	**Sjögren** **syndrome**	**6.8**	**12**	**1752**	20/20; 20/20		Caucasian
17	F	44	RA	6.25	10	1068	20/20; 20/20		Caucasian
18	M	23	SLE	4.8	7	1022	20/20; 20/20		Asian
19	F	23	SLE	3.9	1	146	20/20; 20/20		African American
20	F	17	RA	4.2	5	282	20/20; 20/20		Caucasian
21	F	48	Multiple autoimmune syndrome	3.5	2	146	20/20; 20/20		Caucasian
22	F	55	Lyme disease	3	6	438	20/30; 20/50		African American
23	F	10	Sjögren syndrome	7.4	2	146	20/20; 20/20		Hispanic
24	F	53	SLE	4.4	13	1898	20/25; 20/20		Hispanic
25	F	49	RA	5.9	9	1314	20/20; 20/20		Hispanic
26	F	49	SLE	5.1	17	2482	20/20; 20/20		Hispanic
27^†^	F	73	RA	1.9	26	1460	20/30; 20/25		Caucasian
28^†^	F	78	SLE	1.9	20	1460	20/25; 20/25		Caucasian
29^†^	F	64	SLE	5.4	0.5	84	20/20**^#^**		Caucasian
30^†^	F	64	Sjögren syndrome	2.6	2	146	20/20**^#^**		African American
31^†^	F	75	SLE	5.5	3	438	20/25; 20/20		Caucasian

BCVA, best-corrected visual acuity, OD, right eye; OS, left eye; RA, rheumatoid arthritis; SLE, systemic lupus erythematosus.

* The bold font indicates patients with HCQ toxicity, they are also marked with asterisks.

† Patient with an intraocular lens.

# BCVA, best-corrected visual acuity OD.

Patients were excluded if they had a refractive error greater than ±6.0 diopters spherical or ±2.0 diopters cylindrical, an epiretinal membrane, significant cataract, and evidence or a history of other ocular diseases (e.g., glaucoma, diabetes, age-related macular degeneration, or inherited retinal degeneration).

The study protocol was approved by the institutional review board of Columbia University and adhered to the tenets of the Declaration of Helsinki and complied with the Health Insurance Portability and Accountability Act.

Patients had a comprehensive ophthalmologic examination by one of the authors (S.H.T.) that included a full medical history, best-corrected visual acuity, slit-lamp biomicroscopy, and dilated fundus examination. In addition, the following tests were performed on most patients: standard automated “white-on-white” perimetry 10-2 and/or 24-2 visual field program, SD-OCT, SW-AF, NIR-AF, and mfERG testing. The results for one eye, the right eye, from each patient were included for analysis. When the right eye was not available or had poor imaging quality, the left eye was used. For qAF analysis, the results of one or both eyes, when available, were reported.

### Visual Fields

Automated 10-2 visual field tests (Humphrey Visual Field Analyzer; Carl Zeiss Meditec, Dublin, CA) were obtained using the Swedish Interactive Threshold Algorithm (SITA) standard technique. The total deviation and pattern deviation plots were reviewed for abnormalities by two of the authors (L.A.-Q. and V.C.G.) masked to the patients’ clinical data, mfERG, and SD-OCT results. The following were defined as abnormalities on the 10-2 visual field plots: either the presence of paracentral partial or complete ring scotomas (2°–6° from fixation) or two or more contiguous paracentral points with *P* < 0.01 on the pattern deviation plot.

### mfERG

Twenty-eight of the patients had mfERGs that were performed using the Diagnosys LLC (Lowell, MA) system and following International Society for Clinical Electrophysiology of Vision guidelines.^21^ Pupils were dilated with topical 1.0% tropicamide and 2.5% phenylephrine, and the cornea was anesthetized with 0.5% proparacaine. Subjects were instructed to fixate on a small “X” at the center of an array of 103 scaled black and white hexagons that subtended a diameter of 50°. The mean stimulus luminance was 200 cd/m^2^ with dark hexagons <3 cd/m^2^ and bright hexagons 400 cd/m^2^. An infrared camera allowed the examiner to monitor the position of the pupil during the recording. First-order mfERG responses were acquired using a bipolar contact lens electrode (Burian-Allen; Hansen Ophthalmic, Solon City, IA) and a forehead ground electrode. The first-order kernel mfERGs were analyzed using commercial software. Response densities of the first-order kernel were analyzed by grouping the 103 responses into six concentric rings. R_5_ ring ratios were calculated using the R_5_ ring response as the “reference” and dividing by all other ring response amplitudes (R_1_–R_6_).^22^^,^^23^ The ratios of R_5_ to each of the other rings were compared to 95% confidence intervals (CIs) from 20 control eyes of 20 healthy subjects ranging in age from 18 to 71 years (mean age 42 ± 18 years).

### Fundus Imaging

Following pupillary dilation, SW-AF images were acquired using Spectralis HRA+OCT (Heidelberg Engineering, Heidelberg, Germany) (488 nm excitation). For qAF, the protocol followed previously published guidelines.^24^^–^^27^ Briefly, the Spectralis was equipped with an internal fluorescent reference to adjust for variable laser power and detector sensitivity. The images were acquired in high-speed mode (8.9 frames/s), at a minimum of nine frames (video format), and were saved in nonnormalized mode. For image analysis, mean gray levels were measured in eight circular segments (qAF_8_) at an eccentricity of approximately 7° to 9° from the fovea and qAF values were calculated after gray levels were calibrated to gray levels in the reference and after accounting for the zero-gray level of the laser, refractive error, image magnification, and age-adjusted lens transmission.^28^ For each eye, a qAF value was computed as the mean of the qAF values of the eight segments (qAF_8_). qAF was also compared in superior (qAF_3_) versus inferior-nasal and inferior-temporal macula (qAF_5_) in HCQ-treated patients with and without fundus evidence of maculopathy to compare superior/inferior toxicity.^29^ Comparison was made to 374 eyes of 277 controls (aged 5–60 years) without eye disease^20^ and having the following ethnic composition: 87 whites, 79 Hispanics, 47 blacks, 43 Asians, 6 Indians, and 15 subjects reporting more than one race/ethnicity. Color-coded qAF maps were computed based on pixel-wise transformation of qAF values (WaveMetrics, Lake Oswego, OR).

Horizontal SD-OCT scans (9 × 9; 870 nm; 7 µM axial resolution) were acquired through the macula with the Spectralis HRA+OCT (Heidelberg Engineering) in high-resolution mode with averaging of 100 single scans. The scans were registered automatically to a simultaneously acquired IR-R (820 nm) fundus image.

NIR-AF images were acquired with the HRA2 (Heidelberg Engineering) using the indocyanine-green angiography mode (787 nm excitation, 830 nm emission; 30° × 30° field; sensitivity of 96) after focus adjustment in infrared reflectance mode. To obtain the lowest signal-to-noise ratio for NIR-AF and OCT images, the eye-tracking function was used and averaging of 100 single frames to obtain high-quality images that were saved in nonnormalized and normalized modes. ImageJ (Microsoft Java 1.1.4; National Institutes of Health, Bethesda, MD) was used to analyze the nonnormalized images and plot the NIR-AF signal. Twenty-three subjects (mean age 34 years) without a history of eye disease served as the healthy-eye group. These individuals self-identified as Caucasian (11), Black (3), African (2), Asian (4), and Hispanic (3).

## Results

### Clinical Characteristics

Cohort demographics, duration, and cumulative dose of HCQ therapy are presented in the Table. The study cohort consisted of 31 patients (28 females, 3 males) with a mean ± SD age of 47 ± 16 years (age range: 10–78 years) who had a history of HCQ treatment and were seen after treatment was discontinued (*n* = 3) or who were currently taking HCQ (*n* = 28). Of these patients, 15 (15/31; 13 females, 2 males) were treated with high-dose HCQ (total dose >1000 g; mean age of 50 ± 13 years; age range: 23–78 years) for a mean duration of 163 months (range: 84–312 months) or 13.6 years (range: 7–26 years). Another 16 patients (16/31; 15 females, 1 male) were categorized as having low-dose HCQ (total dose <1000 g; mean age of 45 ± 19 years; age range: 10–75 years). The mean daily dose by body weight was 4.9 mg/kg (range: 1.5–8.9 mg/kg) and mean cumulative dose was 913 g (range: 15–2798 g). Four of the high-dose and one of the low-dose treated patients (mean age of 51 ± 3 years; range: 48–58 years) were diagnosed with HCQ-associated BEM based on fundus examination and corroborated with SD-OCT images, visual fields, and mfERG. Three of these patients (P4, P14, and P15) were tested 30, 7, and 0.5 months, respectively, after HCQ treatment was discontinued. Twenty-six patients were phakic and five were pseudophakic.

### Visual Fields and mfERGs

**Figure 1.**
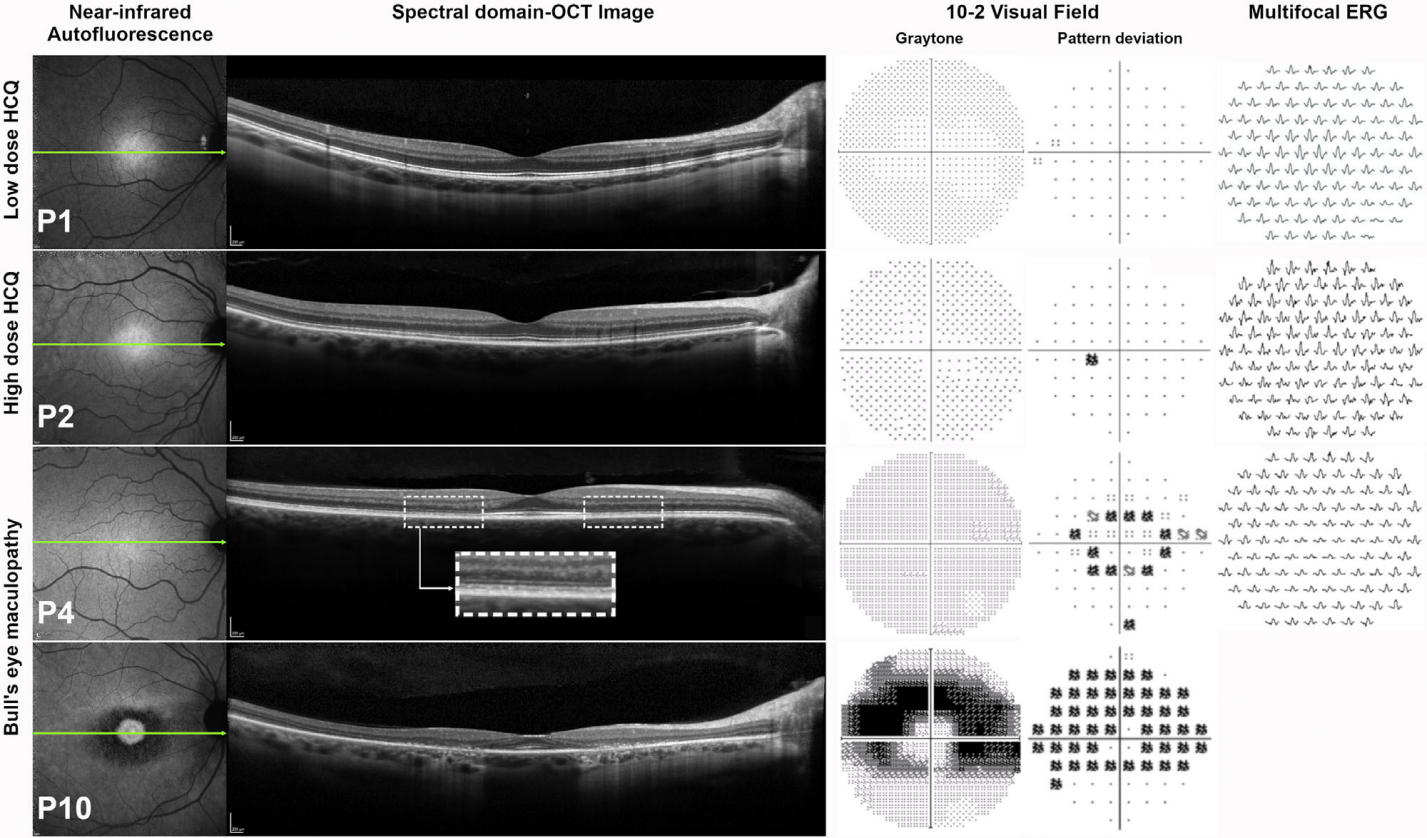
Monitoring the effects of HCQ. Patients (P) 1, 2, 4, and 10. NIR-AF, SD-OCT, 10-2 visual field, and mfERG. P1 (low dose) and P2 (high dose): The NIR-AF image, SD-OCT, 10-2 visual fields, and mfERG were within normal limits. P4 (high dose): The NIR-AF image is unremarkable but the SD-OCT for P4 showed thinning of the outer nuclear layer and disruption of the EZ band in the parafoveal region (*dashed rectangles*). For the 10-2 visual field, a ring scotoma was detected that corresponded to the abnormalities in the SD-OCT image. The mfERG response amplitudes were decreased in the central 10°. P10 (high dose): The NIR-AF image is consistent with BEM. The SD-OCT for P10 showed foveal sparing with loss of the EZ band and RPE atrophy. The 10-2 visual field showed an extensive and pronounced ring scotoma. Note: Left eye of P4 is represented as a right eye.

The visual fields for 24 eyes of 24 patients were classified as normal, six as abnormal, and one as unreliable. Five of the six visual fields classified as abnormal showed parafoveal sensitivity losses with partial or complete paracentral ring scotomas on the 10-2 visual field test, with defects that were more extensive and deeper in the superior field/inferior retina (P4, P10, P14, P15, P16). One patient (P26) had scattered defects in the superior and inferior field. Five of the six patients (P4, P10, P14, P16, P26) with abnormal visual fields had been treated for ≥10 years with a daily dose >5.0 mg/kg, and one (P15) had been treated for 5 years with a daily dose of 4.4 mg/kg. Figure 1 illustrates representative examples of normal (P1, P2) and abnormal (P4, P10) 10-2 visual field results together with the corresponding mfERG trace arrays. For P10, the 10-2 pattern deviation plot shows a central ring scotoma with loss extending more superiorly than inferiorly, and for P4, the 10-2 plot shows a paracentral ring scotoma.

**Figure 2.**
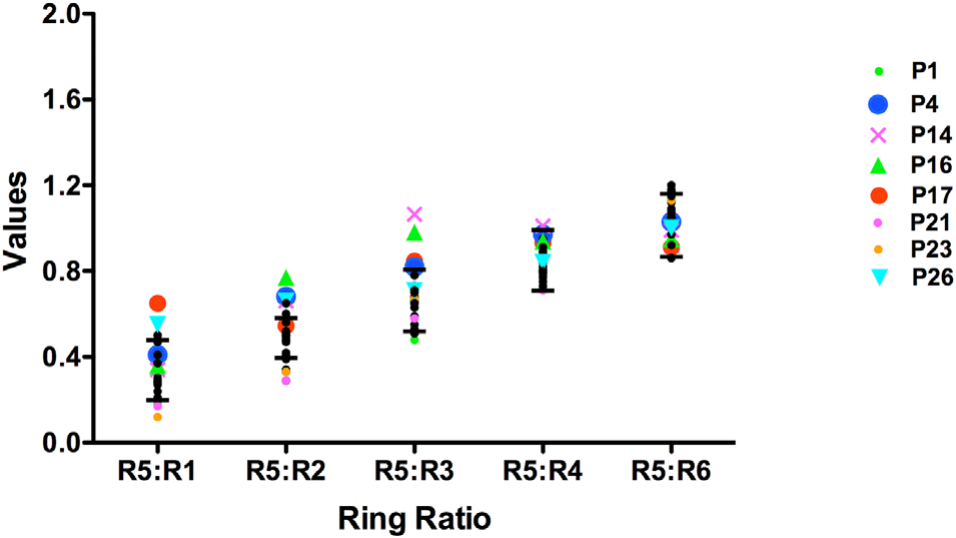
mfERG ring ratio values. R_5_ ring ratio values for 28 eyes of 28 patients. The *colored symbols* represent R_5_ ring ratio values for eight eyes of eight patients with either significantly increased (*n* = 5) or decreased values (*n* = 3); the *horizontal lines* indicate the 5th and 95th percentiles of the normal control values, and the *filled black circles* are the individual values for 20 eyes (20 patients) with normal R_5_ ring ratio values.

Comparisons between the visual field and mfERG results showed that 20 eyes of 20 patients with visual fields classified as normal had mfERG R5 ring ratio values within the normal range, whereas 2 patients with normal fields and 1 with unreliable fields had decreased mfERG ring ratio values (P1, P21, and P23). Significantly increased mfERG R5 ring ratio values were found for one patient (P17) with normal fields. This patient had been treated for 10 years with a daily dose of 6.25 mg/kg. Four of the six patients with abnormal visual fields (P4, P14, P16, and P26) also had significantly increased mfERG R5 ring ratio values, the increased values being consistent with HCQ toxicity.^23^ Significantly increased ratio values were defined as values that were higher than the 95th percentile and significantly decreased values as lower than the 5th percentile. This is illustrated in Figure 2, where the symbols represent R5 ring ratio values for the patients with either significantly increased or decreased values; the horizontal lines indicate the 5th and 95th percentiles of the normal control values and the filled black circles are the individual values for 20 eyes (20 patients) with normal R5 ring ratio values. The greatest differences occurred for the R5:R2 and R5:R3 ratios.

### SD-OCT

Of the 31 patients in the study, visual inspection of the SD-OCT images showed loss or disruption of the ellipsoid zone (EZ) band in five patients (P4, P10, P14, P15, and P16). All five patients showed parafoveal discontinuity or disruption of the EZ band; the “flying saucer sign”^30^ of HCQ retinopathy was evident for P10 and P15. Figure 1 shows the horizontal scans through the fovea for P4 and P10. For P4, there is disruption of the EZ band in the parafoveal region and thinning of the outer nuclear layer indicated by the dashed rectangles. The horizontal scan for P10 is consistent with the “flying saucer sign” and is evidence of severe retinopathy; there is foveal sparing, parafoveal loss of the EZ band, marked thinning of the outer retinal layers, and RPE atrophy. As described above, the five patients had abnormal visual fields typical of HCQ toxicity, and the three referred for mfERG testing (P4, P14, and P16) had significantly increased R5 ring ratio values (Fig. 2). The remaining 26 patients had normal-appearing SD-OCT scans.

### SW-AF

**Figure 3.**
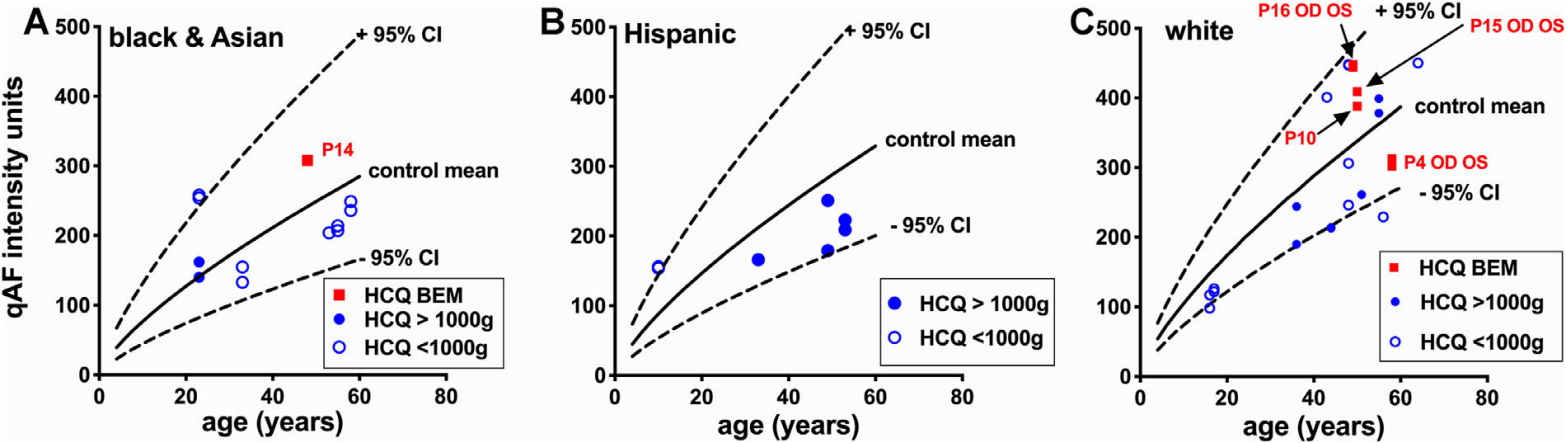
qAF in HCQ-treated patients. qAF8 values acquired as the mean of eight scaled segments positioned at 7° to 9° eccentricity in HCQ-treated patients are plotted as a function of age and ethnicity together with mean (*solid black line*) and 95% confidence intervals (*dashed line*) for healthy subjects. Patients exhibiting BEM (*red circle*; P, patient number) and non-BEM patients exhibiting a cumulative dose of HCQ less than 1000 g (HCQ low dose; *blue open circle*) or a cumulative dose exceeding 1000 g of HCQ (HCQ high dose; *blue filled circle*) are indicated. Symbols for P16 OD, OS and P15 OD, OS are overlapping.

With the exception of four patients (P10, P14, P15, and P16), the patients in the HCQ cohort presented with SW-AF images that were qualitatively unremarkable. SW-AF was measured by qAF using nonnormalized SW-AF images from 31 HCQ-treated patients (51 eyes). Seven eyes from five patients were excluded due to pseudophakia and an age above 60 years; the upper age of our control group is 60 years. For the remaining 26 patients (44 eyes), the qAF8 level of each treated eye was calculated as the mean of intensities determined in each of eight circularly arranged segments (qAF8) situated at an eccentricity of 7° to 9° relative to the fovea. qAF8 is plotted versus age and compared with the 95% CIs of healthy subjects in Figure 3. In the HCQ-treated BEM subgroup (eight eyes, five patients; P4, P10, P14, P15, P16), all qAF8 values were within the 95% CI of healthy eyes stratified for age and race/ethnicity (*P* > 0.05) (Fig. 3). Specifically, of the eight eyes of five patients exhibiting visible BEM, six eyes had qAF8 values (P10 OD, P14 OD; P15 OD and OS; P16 OD and OS) within the upper limits of the confidence intervals, and two were within the lower limits of the confidence intervals (P4 OD, OS).

**Figure 4.**
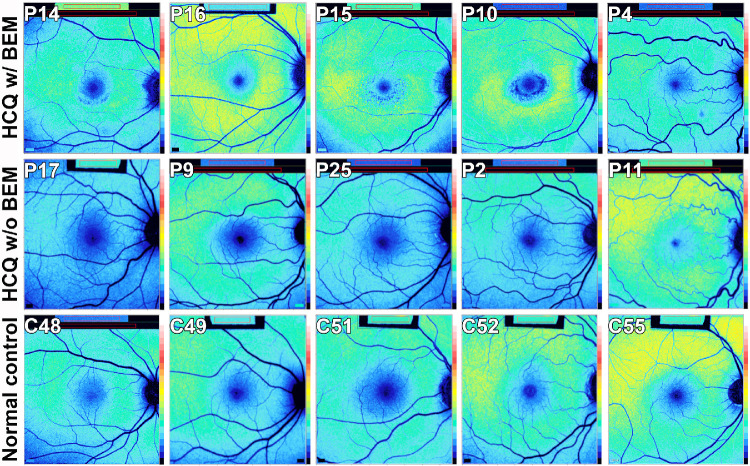
Color-coded qAF images. C, control subject; P, patient. *Top row*: Images acquired from HCQ-treated patients presenting with BEM based on SD-OCT findings. In P10 and P15, note the change in the SW-AF distribution with a higher intensity in the inferior, nasal, and temporal retina than superior retina, as compared to the normal controls (*bottom row*). Mottling is also visible in P10, P15, and perhaps P14. *Second row*: Images from eyes of patients on high-dose HCQ (>1000 g; P17, P25, P2, and P11) and low-dose HCQ (<1000 g, P9). The AF signal appears to have a distribution similar to the control subjects. *Bottom row*: Age-matched healthy eye control. The numbers following the letter C indicate the age of each control subject. The rectangles at the top of the images reflect the internal fluorescent reference used to normalize the intensities. Left eyes are represented as right eyes for P9, P25, and C49.

Color-coded qAF images in healthy eyes exhibit a distinctive pattern (Fig. 4, bottom row). The signal is attenuated in the fovea and perifoveal region due to absorption of the excitation light by macular pigment and due to the increased optical density of melanin. qAF intensities increase with age and are highest in the superotemporal quadrant and lowest in the inferonasal quadrant. Color-coded qAF images acquired from two patients exhibiting BEM (P10, P15) (Fig. 4, top row) revealed a distinct topographic distribution of qAF intensities that differed from the distribution in age-similar healthy eyes. Specifically, in these patients, qAF intensities were noticeably elevated inferior, nasal, and temporal to the fovea. In these two patients (P10, P15), the perifovea exhibited autofluorescence mottling. Less pronounced mottling was detectable inferiorly in P14.

**Figure 5.**
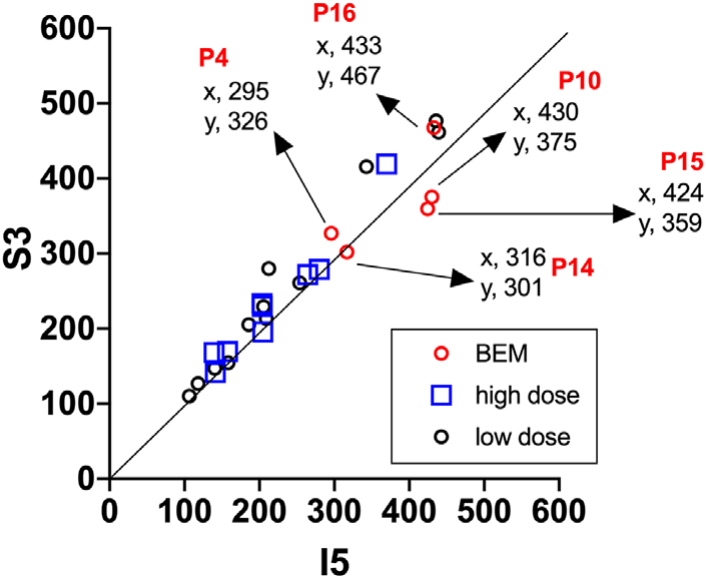
Parafoveal SW-AF intensities in HCQ-treated patients exhibiting BEM. qAF values calculated from SW-AF intensities in eight segments 7° to 9° outside the fovea in patients exhibiting BEM (P4, P10, P14, P15, and P16) and non-BEM patients treated with high- and low-dose HCQ. Mean values determined for the three superior segments (S3) are plotted on the *y*-axis and mean values for the five remaining segments (I5; inferior, nasal, and temporal) are plotted on the *x*-axis. The diagonal line of identity represents *x* = *y*.

To examine for AF asymmetry (Fig. 5) in the patients exhibiting BEM (Fig. 4A: P4, P10, P14, P15, P16), we plotted qAF values for the three superior (S3) qAF segments (*y*-axis) versus the five remaining segments (inferior, nasal, and temporal; I5) (*x*-axis). The data were acquired from the eight segments situated at an eccentricity of 7° to 9° within the measurement grid; at this eccentricity, interference by macular pigment is avoided. Also plotted are the data acquired from high-dose- and low-dose-treated patients. Most data points acquired from the high-dose and low-dose patients were located on or close to the 45° identity line where *x* = *y* (slope 1), indicating that there was good agreement between the qAF intensities in the S3 versus I5 segments. In the case of the five BEM patients, one data set was on the line (P14); the two below the line (P15, P10) were indicative of a superior-inferior asymmetry in qAF. Two other data points were above the line (P4, P16). As shown for P10 in Figure 1, the SD-OCT images acquired from both P15 and P10 also presented with the “flying saucer” sign^30^ indicative of advanced HCQ retinopathy. Note that the abnormal visual fields in P4, P10, P14, P15, and P16 also exhibited superior-inferior differences.

### NIR-AF

**Figure 6.**
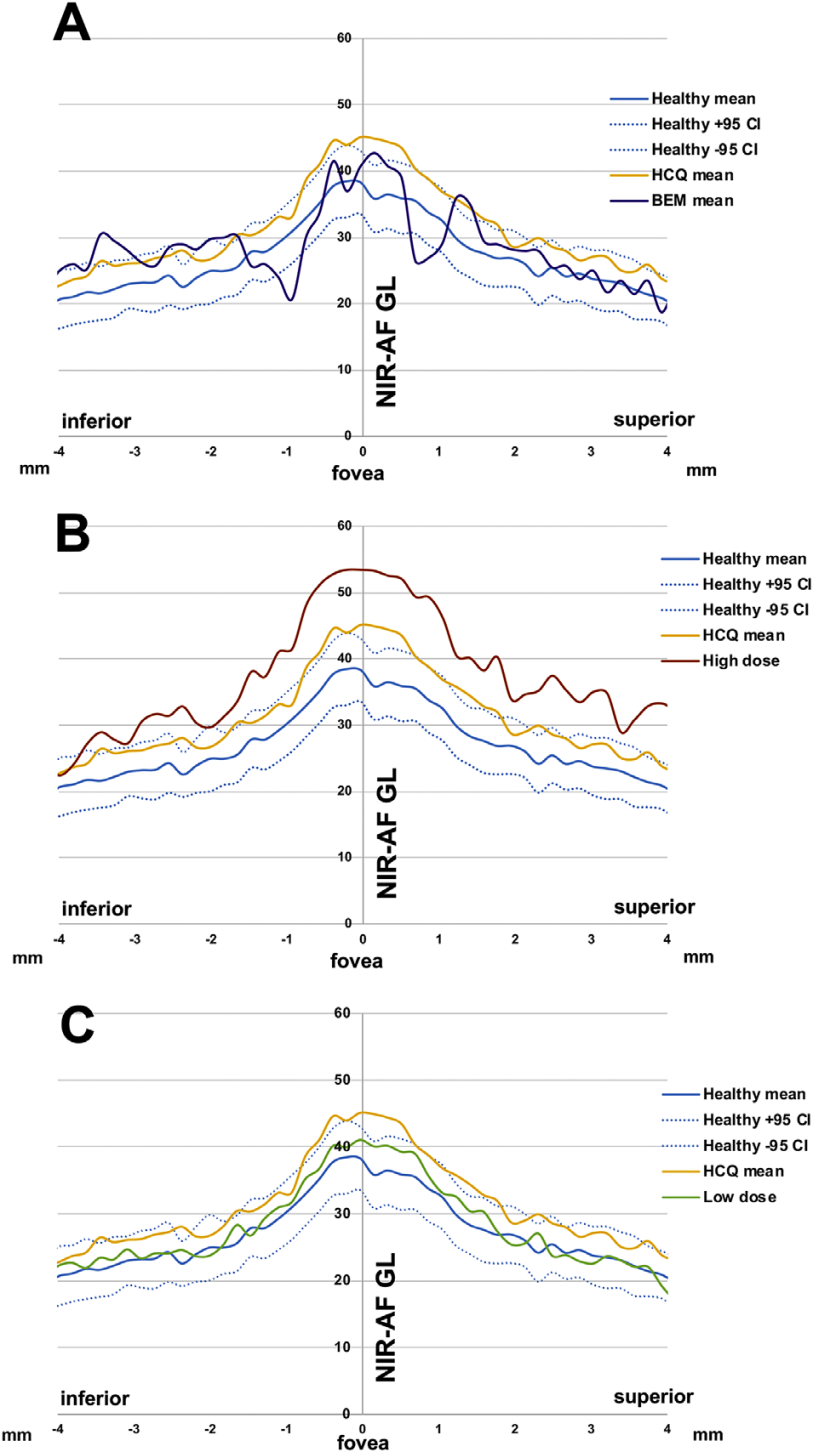
Near-infrared autofluorescence (NIR-AF) profiles measured as gray levels in eyes of HCQ-treated patients. (**A**) Mean (*solid blue lines*) and 95% confidence intervals (*dashed blue lines*) of healthy control eyes compared to mean NIR-AF intensity of 24 eyes of 13 HCQ-treated patients (*yellow line*) and two of these patients (four eyes) exhibiting BEM (*purple line*). (**B**) Mean NIR-AF intensity plotted for four non-BEM patients (eight eyes) treated with high-dose HCQ (*brown line*) together with the mean of all HCQ patients and controls as in **A**. (**C**) Mean NIR-AF intensity plotted for seven patients (12 eyes) treated with low-dose HCQ (*green line*) together with the mean of all HCQ patients and controls as in **A**.

We also analyzed NIR-AF gray-level intensities in 13 patients; 2 exhibited BEM (P4 and P10). The intensities were measured at 47 equally spaced points along a vertical axis through the fovea that extended 4 mm in both superior and inferior directions. Plotting of these NIR-AF profiles demonstrated that the mean NIR-AF intensity calculated from values acquired from the eyes of HCQ-treated patients corresponded to the upper confidence limit (95%) for healthy eyes (23 subjects, 39 eyes) (Figs. 6A–C, yellow trace). For the seven patients/12 eyes (P7, P29, P9, P1, P5, P6, P23) receiving low-dose HCQ (<1000 g), the mean NIR-AF intensity was within the 95% CIs of the healthy eyes (Fig. 6C, green trace) (*P* > 0.05). On the other hand, the mean NIR-AF intensity of four patients/8 eyes (P2, P3, P8, P24) receiving high-dose HCQ (>1000 g) was outside the upper 95% confidence level of healthy eyes (Fig. 6B, brown trace) (*P* < 0.05). In the case of the two patients (P4, P10) exhibiting BEM and from whom NIR-AF images were acquired, the NIR-AF intensity within the parafovea (∼ 1 mm superior and inferior to the fovea) fell below the normal range (Fig. 6A, purple trace) (*P* < 0.05).

## Discussion

HCQ retinopathy typically presents as a parafoveal partial or complete ring scotoma on Humphrey Visual Field (HVF) testing, increased mfERG R5 ring ratio values, and parafoveal loss or disruption of the EZ band in SD-OCT scans. A perifoveal or parafoveal ring of hyperautofluorescence in SW-AF images is also typical. In our cohort of 31 patients, we observed these changes in five patients.

We found that the qAF approach to measuring SW-AF intensities revealed pronounced differences between the superior versus the inferior/temporal/nasal macular areas in two patients exhibiting HCQ-associated BEM. In qAF color-coded images, we noted a change in the topographic distribution of qAF from the normal population with visibly higher signal inferior to the fovea. This difference was corroborated by plotting the data sets with reference to a line of identity where *y* = *x* (Fig. 5). The visual field defects were also deeper and more extensive in the superior field (i.e., inferior retina). Superior versus inferior retinal differences in the progression of other retinal diseases have been reported in both humans^31^ and mouse models.^32^^–^^34^ In many cases, this difference has been attributed to the modifying effects of light. However, regardless of possible explanations, our findings stress the importance of this region when screening for HCQ retinopathy.

Changes in NIR-AF originating primarily in melanin were also observed, although here the differences were not asymmetrical. Thus, NIR-AF intensities measured in the four patients receiving high-dose HCQ (>1000 g) were elevated superior and inferior to the fovea (4 mm eccentricity in both directions). Moreover, in the two patients exhibiting BEM, the NIR-AF intensity within the parafovea dipped (∼1 mm superior and inferior to the fovea) below the normal range.

What are the possible explanations for the aberrant qAF patterning detected in some patients exhibiting BEM? Under several conditions, we have noted that dysfunctional photoreceptor cells can exhibit enhanced bisretinoid production. For instance, within the autofluorescence rings, which often characterize SW-AF images in retinitis pigmentosa,^35^^–^^39^ photoreceptor cell viability is reduced and SW-AF intensity (qAF) can be higher than at equivalent fundus positions in healthy eyes.^40^ Additionally, fundus flecks in recessive Stargardt disease (STGD1) present as hyperautofluorescent foci in SW-AF images while corresponding to hyperreflective lesions that replace photoreceptor-attributable bands in SD-OCT scans.^41^ In acute zonal occult outer retinopathy, qAF is elevated at the border between diseased and nondiseased retina, and at this lesion, border SD-OCT imaging reveals a loss of photoreceptor cell integrity.^42^ Abnormally increased SW-AF is also associated with outer segments that form the core of photoreceptor cell folds or rosettes in degenerating mouse retina,^43^^,^^44^ and in *Mertk*^–^^/^^–^ mice, the process of photoreceptor cell degeneration also involves increased bisretinoid formation.^34^ Similarly, parafoveal increased qAF may signal photoreceptor cell impairment.

It has also been reported that CQ and HCQ inhibit the uptake activity of organic anion transporting polypeptide 1A2 (OATP1A2) that is expressed in RPE and that is purported to transport vitamin A.^45^^,^^46^ Whether this interference impacts bisretinoid lipofuscin formation requires further study.

With regard to the risk of developing retinal toxicity, aside from this being dependent on the daily HCQ dose and the duration of use, genetic factors may also play a role. For example, there are reports that monoallelic variants in the *ABCA4* gene play a role.^47^ The phenotype of HCQ toxicity is similar to cases of *ABCA4* disease (STGD1) that manifest as a BEM.^48^ An National Eye Institute (NEI) clinical trial is currently recruiting patients for a genotype-phenotype study of HCQ-induced retinal toxicity and involvement of the *ABCA4* gene (ClinicalTrials.gov identifier: NCT01145196). As in *ABCA4*-related retinal disease, if an association between *ABCA4* variants and HCQ^47^ were to exist, it may be explained by levels of bisretinoid lipofuscin.^49^ Bisretinoid lipofuscin accumulates with age,^20^ but evidence that patients are at increased risk of HCQ toxicity after age 60 years^3^^,^^47^^,^^50^^,^^51^ has been questioned.^8^

One of the limitations of the study was that subjects older than 60 years were excluded from qAF analysis. We did not include healthy control subjects older than 60 years in our database of qAF values because of reduced ocular media transmission. Another limitation of this study was that our HCQ cohort was not genotyped for *ABCA4*. Given the estimated carrier frequency of ∼5% in the general population, we cannot exclude the possibility that some of the cohort were carrying an *ABCA4* mutant allele. Lastly, the number of HCQ-treated patients proceeding to maculopathy was low, but this was consistent with other reports of HCQ-linked BEM.^7^ The patients presenting with maculopathy based on visual fields and SD-OCT findings were not at the same stage of the disease when qAF imaging was performed. Moreover, high-dose HCQ and the presence of BEM were also associated with changes in the NIR-AF signal originating in melanin. Altogether, the information gained from qAF and NIR-AF has added to our understanding of the mechanisms and sites of HCQ retinopathy.
